# Increased Levels of Eotaxin and MCP-1 in Juvenile Dermatomyositis Median 16.8 Years after Disease Onset; Associations with Disease Activity, Duration and Organ Damage

**DOI:** 10.1371/journal.pone.0092171

**Published:** 2014-03-19

**Authors:** Helga Sanner, Thomas Schwartz, Berit Flatø, Maria Vistnes, Geir Christensen, Ivar Sjaastad

**Affiliations:** 1 Section of Rheumatology, Oslo University Hospital-Rikshospitalet, Oslo, Norway; 2 Norwegian Competence Centre of Pediatric and Adolescent Rheumatology, Oslo University Hospital-Rikshospitalet, Oslo, Norway; 3 Institute for Experimental Medical Research, Oslo University Hospital-Ullevål, Oslo, Norway; 4 KG Jebsen Cardiac Research Center and Center for Heart Failure Research, University of Oslo, Oslo, Norway; 5 Institute for Clinical Medicine, University of Oslo, Oslo, Norway; 6 Department of Cardiology, Oslo University Hospital-Ullevål, Oslo, Norway; University of Leuven, Rega Institute, Belgium

## Abstract

**Objective:**

To compare cytokine profiles in patients with juvenile dermatomyositis (JDM) after medium to long-term follow-up with matched controls, and to examine associations between cytokine levels and disease activity, disease duration and organ damage.

**Methods:**

Fifty-four JDM patients were examined median 16.8 years (2–38) after disease onset (follow-up) and compared with 54 sex- and age-matched controls. Cytokine concentrations in serum were quantified by Luminex technology. In patients, disease activity score (DAS), myositis damage index (MDI) and other disease parameters were collected by chart review (early parameters) and clinical examination (follow-up).

**Results:**

Serum levels of eotaxin, monocyte chemoattractant protein-1 (MCP-1) and interferon-inducible protein 10 (IP-10) were elevated in JDM patients compared to controls (31.5%, 37.2% and 43.2% respectively, all p<0.05). Patients with active (n = 28), but not inactive disease (n = 26) had a higher level of MCP-1 than their respective controls. Levels of eotaxin and MCP-1 correlated with disease duration (r = 0.47 and r = 0.64, both p<0.001) and age in patients, but not with age in controls. At follow-up, MDI was associated with MCP-1(standardized β = 0.43, p = 0.002) after adjusting for disease duration and gender. High MDI 1 year post-diagnosis predicted high levels of eotaxin and MCP-1 at follow-up (standardized β = 0.24 and 0.29, both p<0.05) after adjusting for disease duration and gender.

**Conclusion:**

Patients with JDM had higher eotaxin, MCP-1 and IP-10 than controls. High eotaxin and MCP-1 at follow-up was predicted by early disease parameters, and MCP-1 was associated with organ damage at follow-up, highlighting a role of these chemokines in JDM.

## Introduction

Juvenile dermatomyositis (JDM) is a systemic autoimmune vasculopathy of childhood, involving proximal muscle weakness and characteristic skin lesions. While the mortality rate has decreased (now ∼3%) [1], still 30–61% of patients have signs of sustained disease activity and 60–90% develop organ damage 7.2–16.8 years after disease onset [1]–[3]. Thus new therapeutic targets could improve patient care; however, the pathogenesis of JDM is not fully understood.

Cytokines are small signal molecules, produced by endothelial- immune- and muscle cells. They mediate and regulate innate and adaptive immune responses and inflammatory reactions through a number of mechanisms including recruitment and activation of leukocytes [4]. During the last decade, the role of cytokines and chemokines (chemotactic cytokines) in the pathogenesis in myositis has been an area of interest [5], [6]. However, most studies performed on cytokines consist of mixed patients groups with polymyositis (PM), adult dermatomyositis (DM) and juvenile DM and if controlled, the studies are small. Increased plasma levels of interleukine 18 (IL-18) [7] and IL-15 [8] are reported in patients with DM/PM early in the disease course (first year and median 1 year, respectively). IL-15 was also shown to correlate with disease activity [8]. In a controlled study on 37 DM and 19 JDM patients (median disease duration 2 years), several chemokines including monocyte attractant protein-1 (MCP-1) and interferon-inducible protein 10 (IP-10) were increased [9]. Recently, criteria for clinically inactive disease state in JDM were proposed by the Paediatric Rheumatology International Trials Organization (PRINTO) [10]; however it is not clear whether disease state is associated with a specific signature of cytokines or inflammatory parameters.

Knowledge about cytokine abundance in myositis, in particular JDM, is limited. Specifically there is lack of studies with long-term follow up. Our sex- and age-matched patient-control pairs [11], [12] provide a unique opportunity to compare the cytokine profile in JDM patients after medium to long-term follow-up and to explore how cytokine levels correlate with measures of disease activity and damage at follow-up and at 1 year post-diagnosis.

## Materials and Methods

### Patients and controls

Inclusion criteria were a probable or definitive diagnosis of DM according to the Bohan and Peter criteria [13], disease onset before 18 years, minimum 24 months disease duration and age ≥6 years at inclusion. We identified a retrospective inception cohort of 66 JDM patients diagnosed between January 1970 and June 2006 in Norway, previously described in detail [2], [14], [15]. Four of the patients were deceased; the remaining 62 could all be tracked through the Norwegian population register, and 59 (95%) participated in the study.

Sex- and age-matched controls were randomly drawn from the National Population Register. Exclusion criteria in the controls were: mobility problems, inflammatory rheumatic disease, other autoimmune conditions treated with immunosuppressive agents, and heart or lung disease (except for mild asthma). After cytokine analyses, statistical calculations to detect outliers were performed and 5 pairs were excluded (see statistical analysis). The data presented are based on the remaining 54 patients and 54 controls.

### Ethics statement

Written informed consent was obtained from all patients and controls (and their parents if aged below 16 years), according to the Declaration of Helsinki. The study was approved by the South-Eastern Regional Ethics Committee for Medical Research.

### Data collection and clinical measures

At Oslo University Hospital from September 2005 to May 2009, a single physician (HS) performed clinical examination of all patients, median 16.8 years (range 2–38 years) after disease onset (follow-up), and matched controls. In patients, disease activity was assessed by Disease Activity Score (DAS) for JDM [16] (range 0–20, 0 = no activity), which consists of DAS skin (0–9) and DAS muscle (0–11). Cumulative organ damage was measured by Myositis Damage Index (MDI, range 0–35/40) [17]. In addition, retrospective scoring of DAS and MDI from the first year post-diagnosis were performed, based on chart review [2]. From the criteria proposed by PRINTO (2012), inactive disease was defined as at least 3 of the following 4: manual muscle test (MMT-8) ≥78 (0–80), physician global assessment of muscle activity (phyGloVAS) ≤0.2, Childhood Myositis Assessment Scale (CMAS) ≥48 and creatine kinase (CK) ≤150 [10], [18]. JDM patients with inactive disease are referred to as JDM-inactive and the remaining patients are called JDM-active. Physical health was measured by the Short Form-36 (SF-36) physical component summary score (PCS) [19]. The Health Assessment Questionnaire (HAQ) [20] and the Childhood HAQ [21] were used to measure physical function in patients aged ≥18 years (n = 35) and <18 years (n = 19), respectively. At time of follow-up, none of the study participants had clinical signs of infection. Disease onset was defined as the time of the first muscle or skin symptom clearly related to JDM (by chart review) and disease duration as the time from disease onset to follow-up examination. History of medication was obtained from study cases and by chart review.

### Laboratory analyses

At follow-up examination, venous blood samples were collected and serum concentrations of 29 cytokines analysed. IL-1β, IL-1 receptor antagonist (Ra), IL-2, IL-4, IL-5, IL-6, IL-7, IL-8 (CXCL8), IL-9, IL-10, IL-12, IL-13, IL-15, IL-17, basic fibroblast growth factor (bFGF), granulocyte-colony-stimulating factor (G-CSF), granulocyte-macrophage colony-stimulating factor (GM-CSF), interferon γ (IFN-γ), IP-10 (CXCL10), MCP-1 (CCL2), macrophage inflammatory protein 1α (MIP-1α) (CCL3), MIP-1β (CCL4), eotaxin (CCL11), platelet-derived growth factor bb (PDGF), TNF-α, and vascular endothelial growth factor (VEGF) were quantified using Bio-Plex protein array systems (Bio-Rad, Hercules, CA), based on xMAP technology (Luminex, Austin, TX). The Luminex analyses were performed according to manufacturer's protocol, with minor modifications [22], including selection of high-sensitivity standard curve to optimize measurements of non-septic concentrations of cytokines. However, the high-sensitivity standard curve yielded physiological concentrations of Regulated upon Activation, Normal T-cell Expressed, and Secreted (RANTES/CCL5) above detection limit. RANTES was therefore excluded for further analyses. An intra-assay variation with a coefficient of variation (CV) of 7.49±0.81 was calculated based on measurements of standards. To diminish the effect of the inter-assay variation, all samples were analyzed in a randomized fashion. Three of the 29 cytokines, IFN- α, IL-18 and transforming growth factor β1 (TGF-β1), were analysed with enzyme-linked immunosorbent assay (ELISA) technique.

Along with cytokine analyses, Th1/Th2 cell balance (ratio between CD4+ Th1 helper cells that produce IFN-γ and IL-2 and CD4+ Th2 helper cells that produce IL-4, IL-5, IL-6, IL-10 and IL-13) was evaluated by calculating the ratio of IFN-γ/IL-4 [23]. Erythrocyte sedimentation rates (ESR) were assessed and high-sensitive serum concentration of C-reactive protein (CRP) analysed.

### Statistical analysis

Differences between patients and matched controls were tested by the paired sample t-test for normally distributed continuous variables. Two tailed tests were used for all calculations except for comparisons where a priori patients, based on the literature were likely not to have lower values than controls (e.g. ESR and CRP). Bonferroni correction was performed when appropriate. Correlations were determined by Spearman correlation coefficient (r). Association between eotaxin and MCP-1 (dependent variables) and MDI, DAS skin and DAS muscle measured 1 year post-diagnosis and at follow-up (independent variables) were tested in multivariate linear regression models with forward deletion of the variables after controlling for age and gender. Age was not included in the linear regression model due to high intercorrelation (r = 0.9) with disease duration. p value <0.05 was considered significant. SPSS version 20.0 (SPSS, Chicago, Il) was used for statistical analyses.

To detect outlying individuals, we calculated the mean cytokine levels for all groups and found the Mahalanobis distance from the cytokine level of each individual to its respective group mean. Bonferroni corrected p values were obtained based on an approximation of the Mahalanobis distance to a chi square distribution with the number of cytokines as degrees of freedom. One patient and four controls had samples with a p value <0.001 and were therefore considered to be outliers. These five and their matched control or patient were removed from the data set before the remaining statistical analyses, hence data from 54 pairs were analyzed and presented.

## Results

### Characteristics and serum cytokine levels in JDM patients and controls

Characteristics of the 54 JDM patients and 54 sex- and age-matched controls are shown in Table 1. Eotaxin-, MCP-1- and IP-10-levels were higher in patients than in controls (31.5%, 37.2% and 43.2% respectively, all p<0.05, Table 2). No differences between patients and controls in levels of the other 26 cytokines, Th1/Th2 ratio (Table 2), CRP or ESR were found (Table 1).

**Table 1:** Characteristics and disease parameters in 54 patients with juvenile dermatomyositis and in 54 controls.

Characteristics	JDM patients	Controls
Females	32 (59)	32 (59)
Age at symptom onset (years)	7.7 (1.4–17.3)	NA
Age at diagnosis (years)	8.5 (2.1–19.3)	NA
*Variables assessed median 16.8 years after disease onset (follow-up)*		
Age (years) at follow-up	22.0 (6.7–55.4)	22.1 (6.2–55.4)
Duration from disease onset (years)	16.8 (2.0–38.1)	NA
CRP (<4 mg/L)	2.3 (3.3)	1.4 (3.1)
ESR (<17 mm)†	7.0 (5.7)	5.7 (4.8)
SF 36 PCS‡ (0–100)	54.3 (26.9–60.9)	56.9 (32.1–63.7)*
CHAQ/HAQ (0–3)	0 (0–1.38)	NA
MDI total (0–40)	3 (0–13)	NA
DAS skin (0–9)	4 (0–7)	NA
DAS muscle (0–11)	1 (0–8)	NA
DAS total (0–20)	5 (0–13)	NA
Prednisolone dosis, cumulative (g)	10.6 (12.3)	NA
Prednisolone or DMARDs	16 (30)	NA
*Variables assessed 1 year post- diagnosis*		
MDI total (0–40)	1 (0–7)	NA
DAS skin (0–9)	4 (0–8)	NA
DAS muscle (0–11)	1 (0–7)	NA
DAS total (0–20)	5 (0–15)	NA

Values are number (%), median (range) or mean (SD). JDM: juvenile dermatomyositis; NA: not applicable; CRP: C-reactive protein; ESR: erythrocyte sedimentation rate; SF-36 PCS: Short Form 36 physical component Summary; CHAQ: Childhood Health Assessment Questionnaire; DMARDs: disease modifying anti-rheumatic drugs; MDI: Myositis Damage Index; DAS: Disease Activity Score.

*p<0.05.

‡ n* = *46 pairs, only assessed in those >13 years;

† n = 50 pairs.

**Table 2:** Cytokine levels in patients with juvenile dermatomyositis assessed median 16.8 years after disease onset, and in controls.

	JDM active	JDM inactive	All JDM	Controls	p value
MCP-1	35.5 (19.9)	33.8 (24.2)	34.7 (21.9)	25.3 (11.4)	0.006
IP-10	1598 (1631)	1361 (877)	1484 (1316)	1036 (475)	0.026
Eotaxin	150 (118)	133 (90)	142 (105)	108 (63.6)	0.039
IL-6	8.4 (14.1)	4.9 (4.0)	6.7 (10.6)	4.0 (2.0)	0.060
TNF-α	23.3 (25.7)	21.4 (18.0)	22.4 (22.2)	16.3 (7.2)	0.065
IL-13	2.7 (4.7)	2.8 (4.6)	2.8 (4.6)	1.6 (0.9)	0.078
IL-8	11.3 (3.3)	10.6 (2.0)	10.9 (2.7)	10.2 (2.2)	0.080
IL-1Ra	204 (396)	134 (140)	170 (301)	98.7 (61.5)	0.084
IFN-γ	62.0 (86.8)	49.1 (43.5)	54.7 (70.5)	40.2 (20.9)	0.134
IL-10	6.1 (23.2)	3.5 (6.8)	4.8 (17.3)	1.7 (1.6)	0.183
IL-15	2.3 (3.1)	2.4 (3.0)	2.4 (3.0)	1.7 (1.7)	0.210
IL-18	422 (152)	415 (213)	419 (182)	391 (154)	0.336
TGF-β1	28000 (6770)	29900 (10700)	28900 (8860)	29500 (7360)	0.703
IL-4	2.1 (0.57)	2.1 (0.55)	2.1 (0.55)	2.0 (0.57)	0.794
IL-1β	0.96 (0.90)	1.1 (1.1)	1.0 (1.0)	1.0 (0.9)	0.946
Th1/Th2	27.6 (35.3)	23.8 (22.6)	25.8 (29.6)	19.7 (9.4)	0.780
IFN- α	11.9 (1.8)	11.1 (0.8)	11.5 (1.5)	12.0 (1.4)	0.07

Values for cytokine levels are mean (SD) pg/ml; n: all JDM = 54, controls = 54, JDM active = 28, JDM inactive = 26. p value when comparing cytokine levels in all JDM and controls; for the comparison active *vs* inactive JDM, no differences were detected. The cytokines shown were selected based on associations seen in the present and/or previous studies on dermatomyositis or other rheumatic diseases. JDM: juvenile dermatomyositis; MCP: monocyte chemoattractant protein; IP: interferon-inducible protein; IL: interleukine; TNF: tumor necrosis factor; Ra: receptor antagonist; TGF: transforming growth factor; Th1/Th2, IFN-γ/IL-4.

### Cytokines and inflammatory parameters in JDM-active vs JDM-inactive and in JDM-active and JDM-inactive vs controls

According to PRINTO criteria, 26 (48%) of the patients had inactive disease. No differences were found between JDM-active and JDM-inactive in ESR (8.5 (6.1) vs 5.9 (4.7) mm, p = 0.09), CRP (2.7 (2.8) vs 1.8 (3.7) mg/L, p = 0.36) or in the 29 cytokines studied (Table 2).

However, the 28 JDM-active had 47.9% higher level of MCP-1 (35.5 (19.9) vs 24.0 (10.7) pg/ml, p = 0.012) than their matched controls; between JDM-inactive and their controls, no such difference in MCP-1 levels were seen (33.8 (24.2) vs 26.8 (12.2) pg/ml, p = 0.18.

### Associations between cytokines, age and disease parameters at follow-up

**Figure 1:**
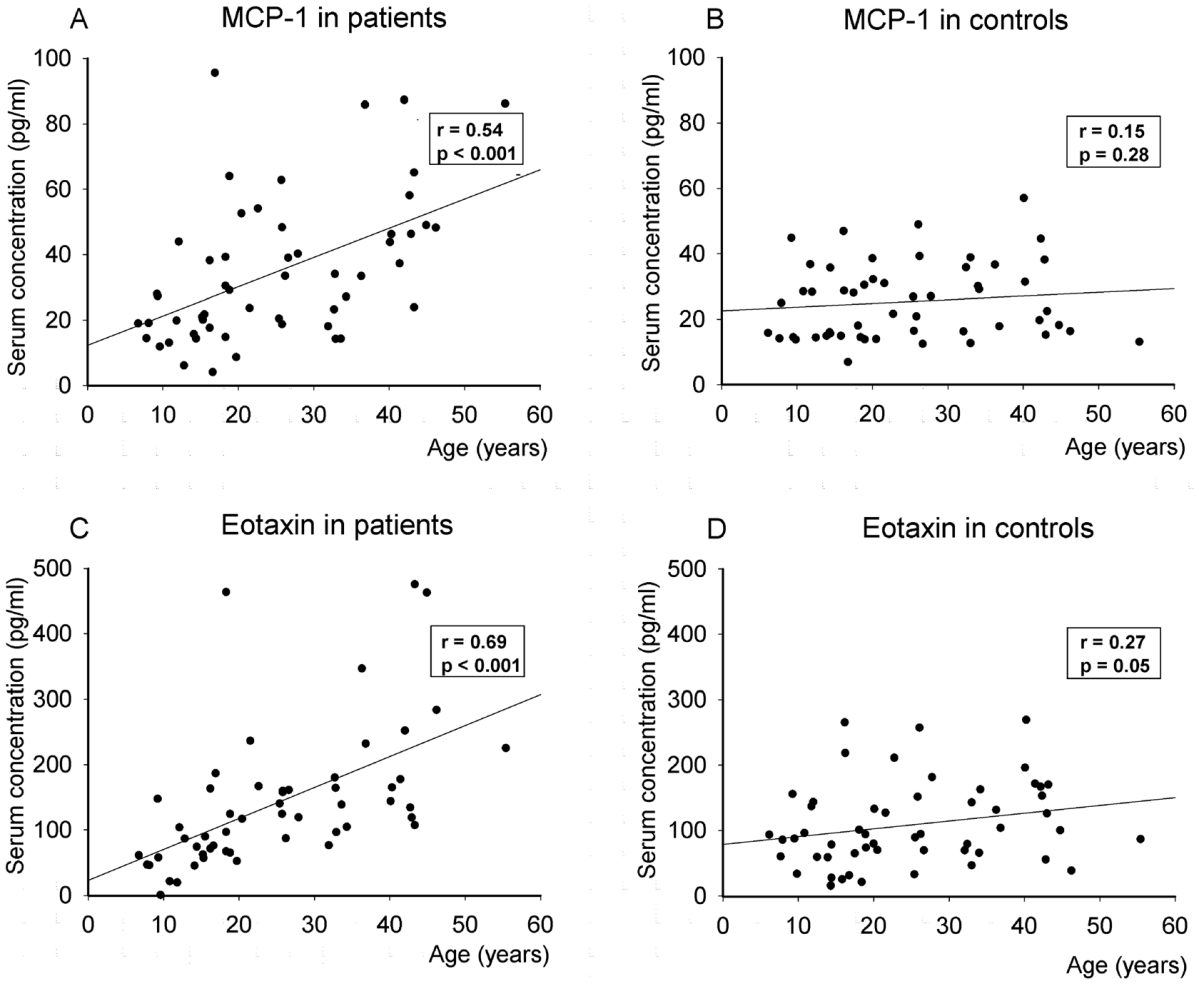
Correlations between monocyte chemoattractant protein-1 (MCP-1) (A and B) and eotaxin (C and D) and age, in 54 patients with juvenile dermatomyositis and sex- and age-matched controls. r, Spearman correlation coefficient.

Eotaxin and MCP-1 both correlated with disease duration (r = 0.64 and r = 0.47, p's<0.001, Table 3 and Figure 1) and age in patients. However, when exploring the association between age and serum cytokine levels in controls, no associations were found. IP-10 correlated neither with disease duration nor with age in patients or controls.

**Table 3:** Correlations between MCP-1, eotaxin and clinical and disease variables in patients with juvenile dermatomyositis assessed median 16.8 years after disease onset, and in controls.

	MCP-1		Eotaxin	
Clinical variables	Patients	Controls	Patients	Controls
Male gender	0.37*	0.21	0.33*	0.39*
Age	0.54**	0.15	0.69**	0.27*
Disease duration	0.47**	NA	0.64**	NA
ESR	−0.05	−0.08	−0.10	−0.20
CRP	0.27*	0.15	0.09	0.01
Eotaxin	0.70**	0.56**	NA	NA
MDI	0.52**	NA	0.52**	NA
DAS total	0.25	NA	0.18	NA
DAS skin	0.17	NA	0.20	NA
DAS muscle	0.20	NA	0.09	NA
Prednisolone	0.28*	NA	0.22	NA
SF 36 PCS	−0.36*	0.09	−0.24	0.11
CHAQ/HAQ	0.32*	NA	0.21	NA

Values are r = Spearman correlation coefficient. MCP: monocyte chemoattractant protein; ESR: erythrocyte sedimentation rate; CRP: C-reactive protein; MDI: Myositis Damage Index; DAS: Disease Activity Score; Prednisolone: cumulative prednisolone dose during disease course; CHAQ: Childhood Health Assessment Questionnaire; SF-36 PCS: Short Form 36 physical component Summary. *p<0.05; **p<0.001.

Both Eotaxin and MCP-1, but not IP-10, correlated with MDI at follow-up (Table 3). High MCP-1 was associated with high CRP, low SF-36 PCS, high CHAQ/HAQ and high cumulative prednisolone dose. Eotaxin and MCP-1 intercorrelated stronger in patients than in controls. No intercorrelation between IP-10 and IFN-α was seen neither in patients nor in controls (r = −0.12 and r = 0.11). No correlations were seen neither between eotaxin, MCP-1 nor IP-10 and disease activity (DAS) at follow-up. In a multivariate linear regression analysis, MDI at follow-up was associated with MCP-1 (standardized β = 0.43, p = 0.002, R^2^ final model  = 40%), none of the control measures (age and gender) were significant. A borderline significant association was seen between MDI and eotaxin (standardized β = 0.25, p = 0.054) in a similar linear regression analysis.

### Early predictors of elevated eotaxin and MCP-1 levels

MDI and DAS total assessed 1 year post-diagnosis, correlated both with eotaxin and MCP-1 (Table 4). DAS skin, but not DAS muscle correlated with eotaxin and borderline with MCP-1.

**Table 4:** Correlations between MCP-1, eotaxin and disease variables in patients with juvenile dermatomyositis 1 year post -diagnosis.

	MCP-1		Eotaxin	
	r	p value	r	p value
MDI	0.35	0.01	0.40	0.003
DAS total	0.28	0.039	0.36	0.007
DAS skin	0.27	0.053	0.36	0.008
DAS muscle	0.18	0.20	0.18	0.21

MCP: monocyte chemoattractant protein; r: Spearman correlation coefficient; MDI: Myositis Damage Index; DAS: Disease Activity Score.

In a linear regression analysis, MDI 1 year post-diagnosis predicted high MCP-1 (standardized β = 0.29, p = 0.025). Of the control measures, disease duration contributed significantly (standardized β = 0.32, p = 0.014), but not gender (R^2^ final model  = 34%).

Accordingly, MDI 1 year post-diagnosis also predicted high eotaxin (standardized β = 0.24, p = 0.049), both of the control measures were significant (gender, standardized β = 0.23, p = 0.045; disease duration, standardized β = 0.41, p = 0.001; R^2^ final model  = 41%).

## Discussion

In our study we have investigated cytokine abundance in JDM patients and found, median 16.8 years after disease onset, increased serum levels of eotaxin, MCP-1 and IP-10, compared to matched controls. When stratified in JDM-active and JDM-inactive, MCP-1 was elevated in JDM-active in comparison to their respective controls; not in JDM-inactive compared to controls. Eotaxin and MCP-1 both correlated with disease duration, and increased levels were predicted by high score of organ damage early in the disease course. MCP-1 was associated with cumulative organ damage at follow-up. To our knowledge, no other controlled study has investigated circulating cytokine profile in an unselected JDM cohort after long-term follow up.

We have previously described the representativeness of our cohort [2], which we believe contains the vast majority of Norwegian JDM patients diagnosed between 1970 and 2006. Our cohort is comparable with other hospital or registry based cohorts with regards to female predominance, age at diagnosis, medication and muscle weakness at disease onset [24], [25]. The representativeness of the patients and the sex- and age matching with controls drawn randomly from the National Population Register, represent strengths of our study.

We aimed at detecting differences in circulating levels of cytokines in JDM patients compared to controls, and found a significant increase in 3 and a numeric increase with p values of 0.06–0.08 for 5 of 28 cytokines. Eotaxin and MCP-1 correlated with disease duration and therefore, necessarily with age. For patients, age was substantially stronger correlated with eotaxin and MCP-1 than for controls, indicating that the correlation between disease duration and CC chemokines is not driven by aging per se. In previous studies on cytokines, DM and JDM patients have been investigated at time of diagnosis or early in disease course [8], [9]. Increased serum levels of eotaxin, MCP-1 and IP-10, were found in a study of 9 JDM patients with clinically active disease [26]. The association between MCP-1 and active disease is supported by our findings: when stratified according to the recently (2012) proposed PRINTO criteria [10], differences in MCP-1 levels compared to controls were seen in JDM-active but not in JDM-inactive. Although we should be careful with our conclusions; we may have been underpowered to detect differences between JDM-active and JDM-inactive. However, the association with disease duration in all patients suggests that eotaxin and MCP-1 may contribute to a sustained inflammation and continue to play a role in JDM throughout the disease course as well.

Several studies suggest a role of IFN-α activity in adult and juvenile dermatomyositis [27], [28]. Since IFN-α was comparable in patients and controls, we did not analyze correlations with disease parameters.

Our observation that eotaxin correlated with early DAS skin, indicates a link between eotaxin and skin affection, in JDM. Some studies associate eotaxin to fibrosis in different tissues as heart, liver and lungs [29]–[31]. In JDM, eotaxin might induce similar tissue fibrosis, either by recruiting granulocytes that release pro-fibrotic substances, or by itself. Furthermore, we found a correlation between eotaxin and organ damage (MDI) at follow-up, and in this context a pro-fibrotic effect could be relevant. The increased eotaxin and MCP-1 levels in patients could support a hypothesis of low-grade sustained inflammation in JDM, contributing to accumulate organ damage as suggested in juvenile idiopathic arthritis [32]. Furthermore, correlation between eotaxin at follow-up, and disease activity and organ damage at 1 year post-diagnosis could indicate that this is a process initiated early in the course of the disease.

It is reasonable to believe that JDM patients have a more widespread and pronounced inflammation at the time of diagnosis than at long-term follow-up. A large study from Sweden in 2010 showed that in rheumatoid arthritis (RA), many cytokines, including eotaxin and MCP-1, were increased even before disease onset, with further increase at the time of diagnosis [33]. In JDM, a small study showed initial inflammation by measuring increased serum level of IL-18 at time of diagnosis; the level then decreased through the first year of the disease [23]. In our study, extensive information about disease course was obtained through data from patients with disease duration ranging from 2 to 38 years. It is noteworthy that none of the cytokines showed a negative correlation with disease duration as one perhaps might expect. Whether there is a continuous increase in eotaxin abundance after the initial active disease, remains unknown. Given the cross-sectional nature of the study, we did not have data on the cytokine levels from the initial years of the disease. One could speculate in a biphasic response: a high initial level of eotaxin, then a decline until, again, a steady climb after 2 years and onwards based on the data in our study. This could be pursued by comparing our long-term results with a prospective study with serial cytokine samples, during the early phase of the disease.

MCP-1 is an attractor and activator of monocytes and T- lymphocytes and is more studied than eotaxin. Besides being an important actor in the immune response, MCP-1 is involved in inflammation, angiogenesis and formation of atherosclerosis [34]. The angiogenetic effect is especially interesting since JDM is a vasculopathy, this could be evaluated by capillaroscopy.

Homology between eotaxin and MCP-1 is 49% and they share 64% of the protein structure [35]; our data also show intercorrelation between the two. Eotaxin is the natural agonist of CC chemokine receptor 3 (CCR3), thus elevated circulating levels of this chemokine may potentially increase the recruitment of CCR3-expressing cells, thereby maintaining chronic inflammation. However, eotaxin has also been shown to be a partial agonist of the receptor CCR2 for which MCP-1 is a full agonist [36]. Thus, eotaxin can partially block MCP-1 effects and could for instance modulate monocyte recruitment in inflammatory condition which is a main effect of MCP-1. Such interactions may well be present in JDM, although this has not yet been studied.

MCP-1 correlated consistently with organ damage and early disease activity and, as well as with other inflammatory parameters such as CRP. Also, the association with cumulative prednisolone dosis is interesting, possibly reflecting longstanding active disease. In the DM/JDM patients studied by Bilgic et al [9], correlation between MCP-1, IP-10, IL-6 and global disease activity was also found the first two years of the disease. In our study, high early organ damage predicted elevated levels of both eotaxin and MCP-1. This suggests that eotaxin and MCP-1 measured at follow-up could be useful biomarkers of disease outcome in JDM; particularly since they are both associated with long-term cumulative organ damage. Also since eotaxin and MCP-1 are up regulated in the acute phase of JDM [26] one could speculate that these cytokines could be early biomarkers of organ damage late in disease course.

Eotaxin and MCP-1 may represent targets for biological treatment in JDM. Anti-CCL2/MCP-1 [37], anti-CCL11/eotaxin (bertilimumab) and CCR3 antagonist [38] are available and potential treatment options. However, effects of cytokines are diverse and complex. For example: in the literature, IP-10 is considered as a type1 interferon (IFN-α) regulated cytokine [27], despite this, we saw no correlations between IP-10 and IFN-α in our study. Furthermore, one study showed no clinical improvement in RA by blocking CCR2 [37], whereas another study on a mouse model of RA surprisingly showed exacerbation of arthritis when CCR2 was knocked out [39]. Thus, it is not obvious whether modulation MCP-1 or eotaxin targets will have beneficial effects in JDM, and interactions at receptor level between eotaxin and MCP-1 can obscure interpretation of the results.

In conclusion; in 54 JDM patients seen median 16.8 years after symptom onset, we have shown higher levels of eotaxin, MCP-1 and IP-10, compared to controls. On a subgroup level, increased MCP-1 compared to controls was seen only in JDM-active, not in JDM-inactive. Both eotaxin and MCP-1 correlated with disease duration and organ damage; for IP-10, such correlations were not seen. It is not clear whether eotaxin and MCP-1 per se cause sustained inflammation and represent possible therapeutic targets. They might also be markers for disease damage as a result of disease activity caused by other unknown mechanisms. Either way, the novel knowledge on these substances can improve insight and treatment modalities of JDM.
